# A Deep Learning-Based Machine Vision System for Online Monitoring and Quality Evaluation During Multi-Layer Multi-Pass Welding

**DOI:** 10.3390/s25164997

**Published:** 2025-08-12

**Authors:** Van Doi Truong, Yunfeng Wang, Chanhee Won, Jonghun Yoon

**Affiliations:** 1 Department of Mechanical Engineering, Hanyang University, 55, Hanyangdaehak-ro, Sangnok-gu, Ansan-si 15588, Gyeonggi-do, Republic of Korea; 2 BK21 FOUR ERICA-ACE Centre, Hanyang University, 55, Hanyangdaehak-ro, Sangnok-gu, Ansan-si 15588, Gyeonggi-do, Republic of Korea; 3 Autonomous Manufacturing & Process R&D Department, Korea Institute of Industrial Technology, 143, Hanggaul-ro, Sangrok-gu, Ansan-si 15588, Gyeonggi-do, Republic of Korea

**Keywords:** multi-layer multi-pass welding, welding monitoring, welding defect inspection, object detection, deep learning

## Abstract

Multi-layer multi-pass welding plays an important role in manufacturing industries such as nuclear power plants, pressure vessel manufacturing, and ship building. However, distortion or welding defects are still challenges; therefore, welding monitoring and quality control are essential tasks for the dynamic adjustment of execution during welding. The aim was to propose a machine vision system for monitoring and surface quality evaluation during multi-pass welding using a line scanner and infrared camera sensors. The cross-section modelling based on the line scanner data enabled the measurement of distortion and dynamic control of the welding plan. Lack of fusion, porosity, and burn-through defects were intentionally generated by controlling welding parameters to construct a defect inspection dataset. To reduce the influence of material surface colour, the proposed normal map approach combined with a deep learning approach was applied for inspecting the surface defects on each layer, achieving a mean average precision of 0.88. In addition to monitoring the temperature of the weld pool, a burn-through defect detection algorithm was introduced to track welding status. The whole system was integrated into a graphical user interface to visualize the welding progress. This work provides a solid foundation for monitoring and potential for the further development of the automatic adaptive welding system in multi-layer multi-pass welding.

## 1. Introduction

Multi-layer multi-pass (MLMP) welding is a widely used method in various manufacturing industries such as shipbuilding, nuclear power plants, or pressure vessels for constructing durable, high-strength joints of mid-thick materials [1]. MLMP welding involves depositing multiple layers of weld metal until the target groove is filled. Metallurgically, it offers benefits such as grain refinement and more uniform phase segregation, which may reduce thermal cracking [2]. It is particularly effective for filling large grooves or gaps, which helps to minimize common welding defects such as incomplete penetration, lack of fusion, and undercutting. Moreover, it provides flexibility for designing a sequence of welding paths as well as welding parameters. However, MLMP welding also presents several challenges, such as distortion rate accumulation and welding defects, which can considerably reduce the process efficiency and affect product quality.

Distortion is a common problem due to the thermal effect during MLMP welding, which accumulates with every single bead. Therefore, weld seam tracking plays a critical role in controlling the welding torch position as well as dynamically modifying the welding parameters based on distorted weld geometry. Several techniques are used for tracking the weld bead, such as vision sensing, arc sensing, and infrared sensing techniques. Ye et al. [3] proposed a linear-based polynomial regression method to segment the bead parameters, including width and height, based on the laser-line profile sensor data. The author mentioned the problem of bead recognition errors due to the unstable movement of the laser sensor during the welding process. Zhu et al. [4] proposed the use of a local pattern recognition (LPR) algorithm to detect the groove edge position based on infrared visual sensing. The author mentioned that LPR can obtain higher accuracy than the mean algorithm, although it is time-consuming. Yang et al. [5] proposed the edge groove detection method using a multi-layer convolutional neural network with weld images as input. The error of the prediction results is typically less than 0.45 mm, but it can be 0.75 mm when the groove has extreme noise from the welding arc. Mao et al. [6] proposed an integration approach of the elastic finite element method, artificial neural network (ANN), and inherent strain method (ISM) to predict deformation. The ISM can predict the angular distortion of simple bead-on-plate joints under different plate thicknesses and heat input conditions. Liu et al. [7] proposed a restoration and extraction network based on a conditional generative adversarial network to extract seam feature points in MAG welding. The model achieved good results in some initial passes; however, errors increased as the number of passes increased. Therefore, online monitoring of the welding geometry enables dynamic control of the welding path sequence, which can reduce welding defects.

Depending on the material, welding type, and welding condition, various welding defects can occur, such as incomplete penetration, porosity, undercut, crack, slag inclusion, and burn-through [8]. Therefore, welding quality plays an important role because it affects the life cycle of products and safety hazards. Based on sensor characteristics, weld defect inspection can be simply categorized into vision-based and signal-based approaches. The vision-based approach uses vision sensors, such as a Red–Green–Blue (RGB) sensor [9], Grayscale sensor [10,11], line scanner [12], infrared sensor [13], and a radiographic sensor [14], to capture an image and perform image processing to determine the welding defect position. Zhang et al. [9] captured images from three different angles, including the top front, top back, and back seam, and applied the CNN classification model to identify defects. Shafeek et al. [14] presented a vision system to detect gas pipeline defects from radiographic films. The method can detect welding defects in gas pipelines welded by shielded metal arc welding. Bacioiu et al. [11] also proposed a defect detection algorithm for aluminum TIG welding using a high dynamic range (HRD) camera and an ANN model. The signal-based approach collected signals by sensors such as audio or acoustic emission sensors and analyzed them to find defect locations. Luo et al. [15] applied an ANN model using audible sound to diagnose abnormal defects. The wavelet analysis results showed a decrease in the intensity of frequencies below 781 Hz in defective situations. Zhang et al. [16] employed linear and non-linear ultrasonic testing to measure weld morphology, demonstrating a high correlation between ultrasonic energy and weld morphology. Zeng et al. [17] used the fast discrete sine transform (FDST) method and laser ultrasonics to detect weld defects. The study also noted that the FDST method can evaluate the size of weld defects through spectrum images, in comparison to the Fast Fourier Transform method.

Some researchers have proposed seam line tracking and welding defect detection for single-bead welding [3,11,18,19,20,21,22]. However, one of the key challenges in machine vision for welding is the substantial noise interference resulting from arc brightness, which can obscure critical visual information. Additionally, high reflectivity caused by variations in material surface and groove shape can make it difficult for a camera to capture precise data. While acoustic sensors offer a potential method for defect detection, their performance may be compromised by ambient environmental noise. Furthermore, distortion rate accumulation, burn-through, or defect detection, which can significantly impact the efficiency and productivity of MLMP welding, have received less attention.

In this study, novel data processing and analysis methods are developed and validated for monitoring and defect detection in the MLMP welding process. The primary objective is to enable real-time monitoring and analysis of weld bead geometry and temperature, as well as the inspection of surface defects. To achieve this, the study focuses on the following specific aims.

‑ To integrate a line scanner and an infrared sensor into the welding system for the acquisition and visualization of weld bead geometry and thermal data during MLMP welding.
‑ To develop a robust algorithm for detecting groove profiles, modelling weld cross-sections, and reconstructing the three-dimensional welded surface to monitor welding progress.
‑ To develop a processing method for depth data to create input data in a deep learning model used in surface quality evaluation.
‑ To develop an image processing algorithm based on infrared images for burn-through defect inspection during welding.

## 2. Experimental Setup

**Figure 1:**
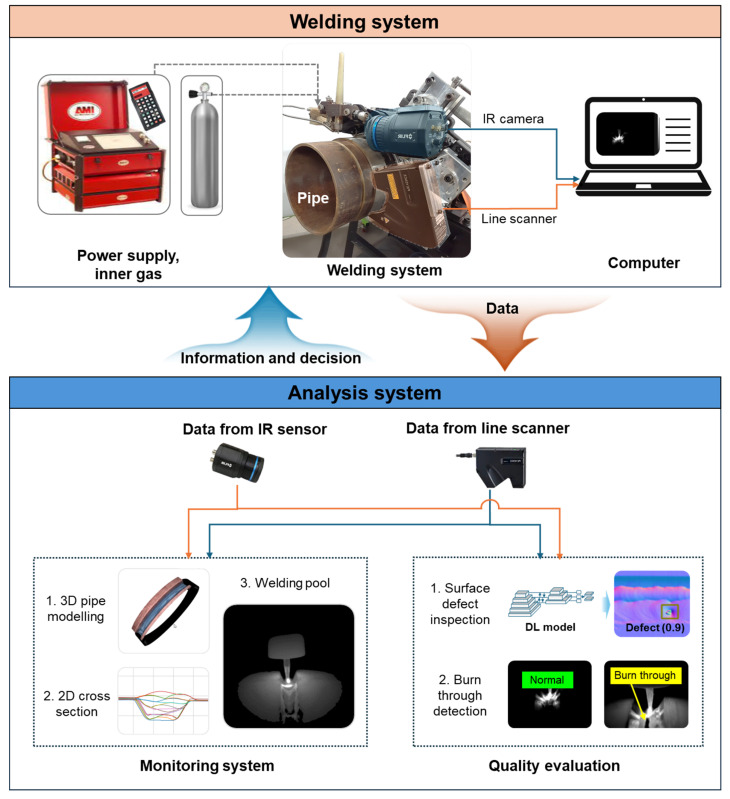
Schematic diagram of the machine vision system for monitoring and quality evaluation during MLMP welding process.

The monitoring system was developed, including two synchronized stages: the first stage was developed for collecting data from the welding system and the second stage was developed for analyzing the collected data, as shown in Figure 1. The welding system consists of a welding machine, power supply, shielding gas, and sensors attached to the welding head and connected to an analysis computer. During welding, the data are collected automatically and transferred to an analysis model. After analyzing a welding pass, the weld bead status is visualized on a user interface, allowing the user to make decisions and control the real welding system based on the analysis results.

To collect data and information from the welding process, two sensors were used: a line scanner and an infrared (IR) camera. The orbital welding head and power supply are programmable, enabling the user to create, save, or modify welding parameters such as arc current, voltage, wire feed speed, travel speed, and welding time for each pass. The wire feeder system automatically feeds the wire based on the setup speed, and the clamping mechanism allows the welding head to move around the steel pipe on the guide rail. The specifications of the experimental equipment are shown in Table 1. The IR camera and line scanner were attached to the welding head, as shown in the welding system in Figure 1. During the welding process, the camera was placed 150 mm from the electrode tip at an 85-degree observation angle and focused on the weld pool, enabling real-time recording, while the line scanner scanned the surface of the welded surface. Due to its mounting position relative to the welding tip, a slight delay in surface data acquisition was introduced. However, as surface defect inspection was conducted after each welding pass without the need for real-time processing, this delay did not impact the welding process.

Multiple MLMP welding experiments were conducted on steel pipes for data collection, with each case consisting of 9 to 12 passes. The detailed information of two common materials is shown in Table 2. The welding conditions were selected based on suggestions of welding experts and intentionally modified to obtain welding defects such as a lack of fusion, porosity, or burn-through. For instance, increasing the welding travel speed was used to create a lack of fusion defects, raising the heat input to make burn-through, and reducing the shield gas flow rate to increase the likelihood of porosity defects.

Regarding the welding sequence, a welding sequence planning algorithm based on parallelogram and trapezoidal shapes was applied. The main principle of the algorithm is the equal welding area assumption. The reason behind this assumption was to reduce the welding parameter modification during the welding process. First, a root pass was in a centre position for filling the gap as well as connecting two pipes. After that, multiple passes were added until the whole groove was filled. Finally, two or three cap passes were added to cover the groove surface.

## 3. Methodologies

**Figure 2:**
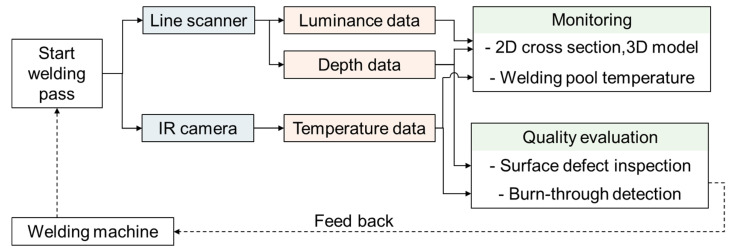
Workflow of the integrated monitoring system with welding defect inspection.

Figure 2 shows the overall procedure of the proposed integrated system consisting of two main targets: monitoring and quality evaluation. During welding, while the line scanner acquired the profile data, the IR camera captured the temperature image focusing on the weld pool. Our proposed groove and weld bead detection algorithms determined the region of interest and the cross-section, whereas the 3D weld bead surface was reconstructed based on the travel speed. In addition, the surface defects were inspected by using the normal map of depth data and the deep learning model. The thermal data were used for monitoring the weld pool as well as burn-through defect detection. If a burn-through problem occurs during welding, the system returns the signal for the welding machine. This section presents detailed descriptions of the four main algorithms, including groove profile detection (Section 3.1), cross-section and welded surface modelling for multi-bead monitoring (Section 3.2), surface defect inspection (Section 3.3), and weld pool monitoring and burn-through defect detection (Section 3.4).

### 3.1. Groove Geometry Detection

**Figure 3:**
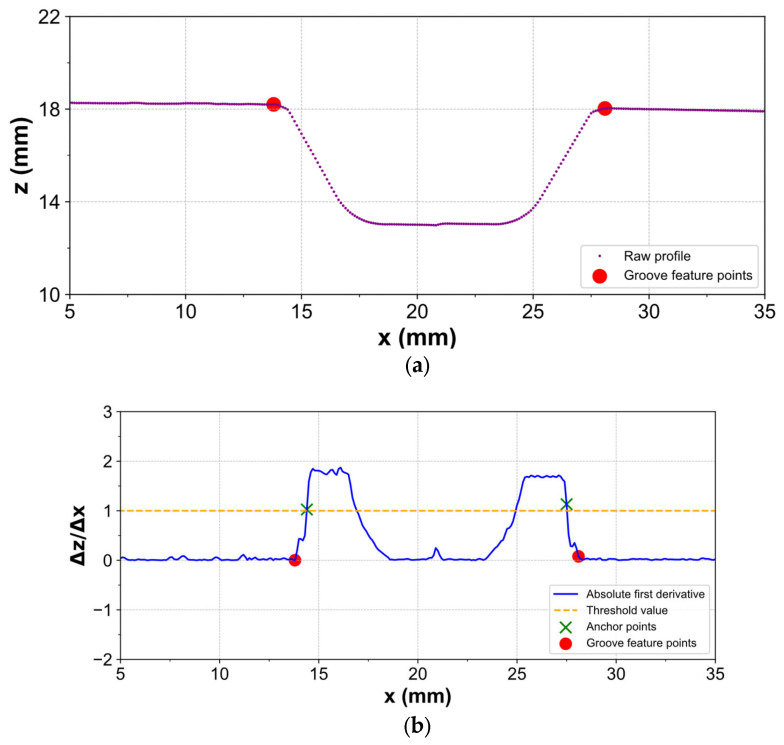
Visualization of groove geometry extraction: (**a**) the groove feature points on the raw profile; (**b**) anchor points and groove feature points by applying a threshold value on the absolute first derivative of the raw profile.

Groove geometry plays a crucial role in determining the region of interest of the welding monitoring location. It is also important information for welding path planning. Before welding, the line scanner was used to scan an initial groove surface. Based on the raw data, the region of interest was determined by the groove extraction algorithm. The pseudo-code of the groove geometry extraction algorithm is shown in Algorithm 1. The raw data are shown in Figure 3a, where the horizontal axis shows the position of the transverse direction and the vertical axis shows the height. It is assumed that the height is a function of x called f(x), and f(x) always has continuous derivatives in all the scanning data domains. Based on a Taylor series for f(x), the first derivative of f(x) at $x\;=\;x_{i}\;$ can be approximated in the second order by Equation (1):

(1) $$f^{\prime }\left(x_{i}\right)\;\approx \;\frac{f\left(x_{i\;+\;1}\right)\;-\;f\left(x_{i\;-\;1}\right)}{2∆x}\;+\;O(∆x^{2})$$

where Δx is the resolution in the x direction of the line scanner profile data, and $f\left(x_{i\;+\;1}\right)$ and $f\left(x_{i\;-\;1}\right)$ are the height values before and after the position $x_{i}$, respectively.

These first derivative values are also the slope at each position on the profile. The line scanner was nearly perpendicular to the groove surface, and the base material surfaces on the left and right sides were nearly parallel. Therefore, both the left and right base materials had similar slopes and are near zero. That was the main feature of setting a threshold value for determining the groove position. Then, the derivative profile was converted to the absolute value, and the threshold value was applied to find two anchor points on the left and right. From the left anchor points, the backward loop was applied to find the left feature point, while the forward loop was used to find the right feature points, as shown in Figure 3b. Finally, two groove feature points were obtained, and the raw profile can be extracted, as shown in Figure 3a. Because two pipes have the same diameter and the pipe’s ends were filled at the same angle, the electrode tip position for welding a root path is the centre of two feature points. Then, the other passes were welded until the whole groove was filled based on the welding sequence plan.
**Algorithm 1:** Algorithm of Proposed Pseudo-Code to Groove Geometry Extraction.
**Input:** Line scanner profile
**Output:** Groove geometry1Get the height and x value from the raw line scanner profile2Compute the first derivative of the line scanner profile3Determine the base material part on the left and right sides based on the slope value4Determine the groove geometry

### 3.2. Cross-Section and Welded Surface Modelling for Multi-Bead Monitoring

**Figure 4:**
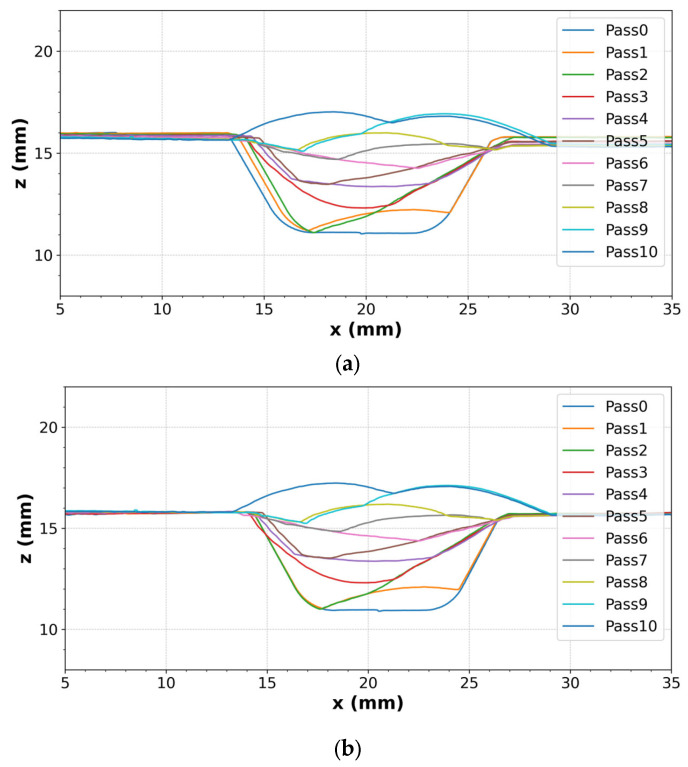
An example of representative cross-section data taken from the line scanner: (**a**) raw data with the accumulated distortion in some initial passes because of the thermal effect; (**b**) the cross-section geometries after alignment.

**Figure 5:**
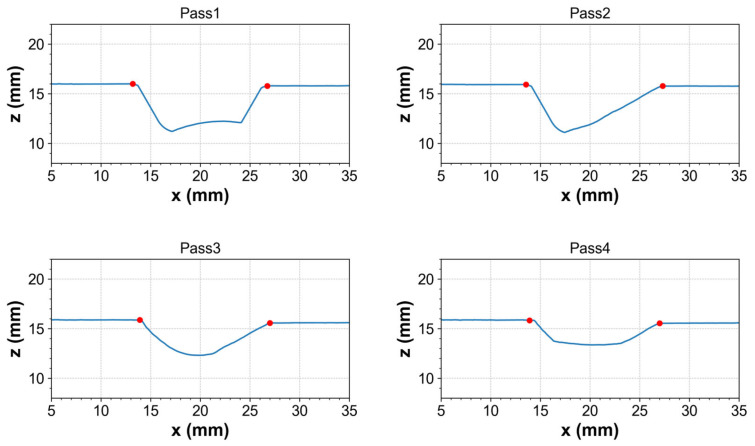
Example of representative groove feature points extracted on the raw profile of four weld beads.

During the welding process, the heat input transferred to the weld pool generates high temperature gradients in the pipe. The thermal effect of welding causes expansion and contraction, leading to a change in the shape or dimension of workpieces, which is referred to as distortion. This distortion tends to accumulate after each welding pass, and the cross-section of the welded seam can also be different compared to the welding planning. Therefore, the seam line should be tracked after each welding pass for dynamic control of the welding position, and the cross-section can be modelled to measure the distortion caused by the thermal effect. To reconstruct the welding section, every single welding pass geometry was extracted and aligned based on its previous pass. Figure 4a shows the raw data of the cross-section with multiple welding pass profiles captured by the line scanner. The effect of distortion or some other environmental conditions can cause small differences between a previous profile and a new profile at positions without welding. To reconstruct the cross-section, the proposed method described in Section 3.1 was first applied to each weld profile to identify the groove feature points that represent consistent landmarks along the weld profile, as shown in Figure 5. During welding, thermal distortion can cause slight shifts in these points, meaning they no longer coincide exactly between profiles. To address this, it was assumed that the two groove feature points maintained a fixed distance and were used as reference points to align all profiles. This assumption enabled consistent alignment across welds, helping to reduce differences in the non-welded areas caused by deformation, thereby reducing differences in the non-welded regions. Consequently, the previous geometries were scaled and aligned to the newly welded profile based on these reference points. The aligned cross-section results are shown in Figure 4b with reduced distortions. Regarding the welding surface modelling, all the welding profile data obtained in each welding pass were gathered, and each point of the welded surface was calculated based on the travel speed and welding time. The 3D structural mesh was generated by connecting 4 points as quadrilateral elements. The results allowed users to interact on a graphical user interface (GUI).

### 3.3. Surface Defect Inspection Using the Line Scanner

**Figure 6:**
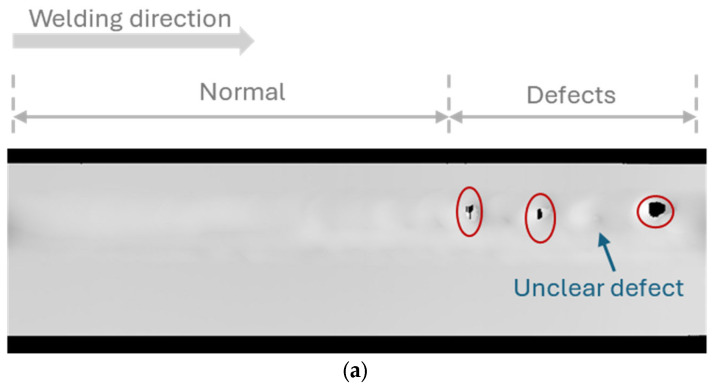

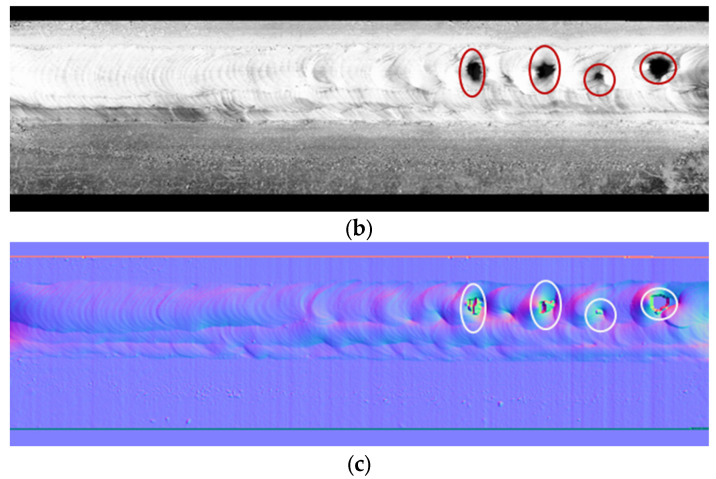
Visualization of welding surface by multiple data types: (**a**) raw depth image, (**b**) raw luminance image, and (**c**) normal image converted from depth data.

During welding, many types of defects can occur, such as a lack of fusion, porosity, and undercut. In addition to the reconstruction of the 3D geometry of weld beads, line scanner data can be used to evaluate the welding surface quality. Two types of raw data, including depth and luminance, were collected from the line scanner, as shown in Figure 6a and b, respectively. In this example data, the depth image and luminance image were generated by combining multiple profiles along a welding direction and observing from the top view. Based on the welding direction from left to right, a normal welding status can be observed on the left side, while multiple defects occurred on the right side. The depth image was not clear enough to see the defect positions as the welding boundary of each pass, while the luminance image can show defects clearly. It is hard to highlight the weld bead because the grayscale value depends on the surface colour and light conditions. Therefore, to identify defect positions and monitor the weld bead on the welded surface, the use of a normal map computed from the depth data was proposed.

It was assumed that the depth map is a function of the x and y coordinates called z = f(x,y). The normal vectors at an arbitrary position ($x_{i},\;y_{i})$ were determined as follows.

(2) $$\vec{n}\;=\;\left[\begin{array}{c} f_{x}^{\prime }(x_{i},\;y_{i})]\\ f_{y}^{\prime }(x_{i},\;y_{i})\\ -1 \end{array}\right]$$

where $f_{x}^{\prime }$ and $f_{y}^{\prime }$ were the gradients of the depth map in the x and y directions, respectively. To compute $f_{x}^{\prime }\;$ and $f_{y}^{\prime }$, the Sobel algorithm was used with the 3 × 3 convolution kernels for the x and y directions as follows.

(3) $$K_{x}\;=\;\left[\begin{array}{ccc} -1 & 0 & 1\\ -2 & 0 & 2\\ -1 & 0 & 1 \end{array}\right],\;\;{\;K}_{y}\;=\;\left[\begin{array}{ccc} -1 & -2 & -1\\ 0 & 0 & 0\\ 1 & 2 & 1 \end{array}\right]\;$$

**Figure 7:**
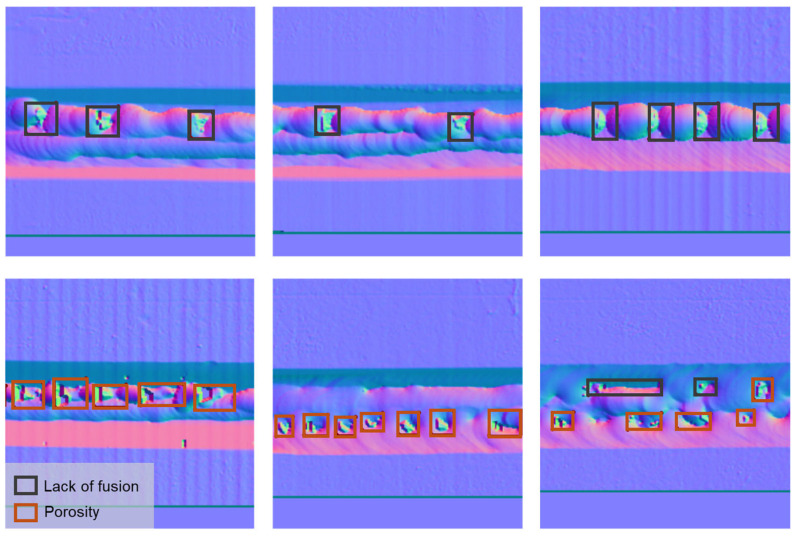
Example images annotated with labels indicating a lack of fusion and porosity defects.

After converting, the normal image was obtained, as shown in Figure 6c, which shows the weld bead and defect position clearly. Due to the variety of defects, a deep learning approach was proposed for defect inspection instead of the conventional image processing method. Recently, object detection using deep learning has been a significant achievement in many industrial applications [23,24,25]. There are two types of approaches: two-stage approaches, such as Fast RCNN, SPP net, and FPN, and a one-stage approach, such as the Yolo family series [26] and SSD. In this study, the YOLOv10 version [27] was used because it was state of the art in object detection with robust performance, fast processing, and high accuracy. Traditionally, other studies used X-ray or RGB images for defect detection. In this study, the normal map derived from depth data was proposed as the input data. The normal map is not only used to eliminate the effects of material colour but also to highlight the weld bead and defect clearly. Regarding the training process, numerous experiments were conducted under different welding conditions to collect diverse data. Then, the raw data were sequentially converted to a normal map of 256 × 256 pixels, corresponding to a real scale of 40 × 40 mm. Two main defects, including a lack of fusion and porosity, were labelled as one defect label only by the bounding box using a LabelMe open-source code [28], as shown in Figure 7. Then, the label was converted to a Common Object in Context (COCO) format, which was usually used for training the deep learning model. The Ultralytics package [29] was used for training, and the metrics outputs were used for evaluating the trained models. The hyperparameters adjusted for training are shown in Table 3. In the testing phase, separate experiments were conducted to evaluate the model on a test dataset.

### 3.4. Weld Pool Temperature Monitoring and Burn-Through Detection Using an IR Camera

**Figure 8:**
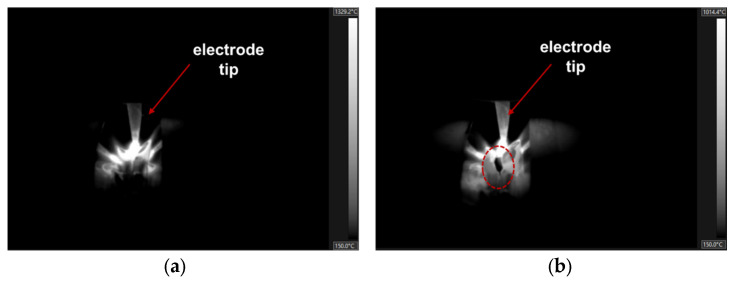
Weld pool captured by an IR camera: (**a**) the weld pool under normal status and (**b**) the weld pool within the burn-through defect.

**Figure 9:**
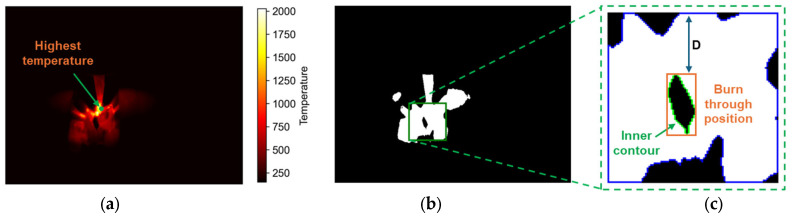
Illustration of the burn-through defect detection algorithm: (**a**) the highest temperature position to determine ROI, (**b**) the binary image after applying the threshold, and (**c**) the burn-through position in the ROI.

The IR camera was used to monitor the weld pool temperature and detect burn-through during welding. Burn-through is an unexpected problem [30] when the base metal is melted completely and creates an undesirable open hole. This can be caused by excessive heat input, such as high amperage, slow travel speed, improper joint penetration, extreme gap, and insufficient metal cleaning. Typically, this defect occurs right on the weld seam, and it commonly occurs during the root pass of MLMP welding because the gap is not welded yet. To evaluate this problem during welding, an image processing algorithm was proposed for burn-through detection. Figure 8 shows the difference between normal welding and burn-through images captured by the IR camera. Compared to the normal image, a hole can be observed at the bottom of the electrode tip in the burn-through image. This hole was considered the main target of the proposed algorithm. The details of the algorithm are shown in Algorithm 2. To identify the electrode tip position, the highest temperature position was found in the temperature map, as shown in Figure 9a, because of the plasma that forms between the electrode and workpieces when an electric current flows through the medium. Because the burn-through always occurs below this position, a region of interest (ROI) was created in the area underneath. The hole usually had a lower temperature than the surrounding area. Therefore, the thresholding method was applied to separate the temperature map into two levels, as shown in Equation (4).

(4) $$\left(x,y\right)\;=\;\left\{\begin{array}{c} 0,\;\;\;t\left(x,y\right)\;<\;T\;\;\\ 255,\;\;t\left(x,y\right)\;>\;T \end{array}\right.\;$$

Here, $t\left(x,y\right)$, g(x, y), and T are the input temperature map, output binary image, and temperature threshold value, respectively. The output binary image is shown in Figure 9b, while the cropped ROI, including the burn-through hole, is shown in Figure 9c. Because the hold was inside a high-temperature area, the hierarchy of contours was used to find the inner contours. If an inner contour exists, the minimum vertical distance (D) between the inner contour and the top edge of ROI was measured and then compared to a certain threshold to reduce the abnormal noise. Finally, the weld pool status was returned as normal or burn-through for visualization.

**Algorithm 2:** Algorithm of Proposed Pseudo-Code to Burn-Through Detection.
**Input:** Temperature data
**Output:** Burn-through or normal status1Find the coordinates of the maximum temperature 2Define a region of interest (ROI) centered at that coordinate 3Apply a threshold to the ROI to create a binary image4Find contours in the binary image5Use contour hierarchy to identify inner contours 6**If** inner contours exist: 7
Measure the minimum vertical distance from the inner contour to the top edge8
**If** the distance < threshold distance:9

**Return:** Burn-through 10
**Else**11

**Return:** Normal12**Else**13
**Return:** Normal

## 4. Results and Discussion

### 4.1. Multi-Pass Weld Bead Modelling Results

**Figure 10:**
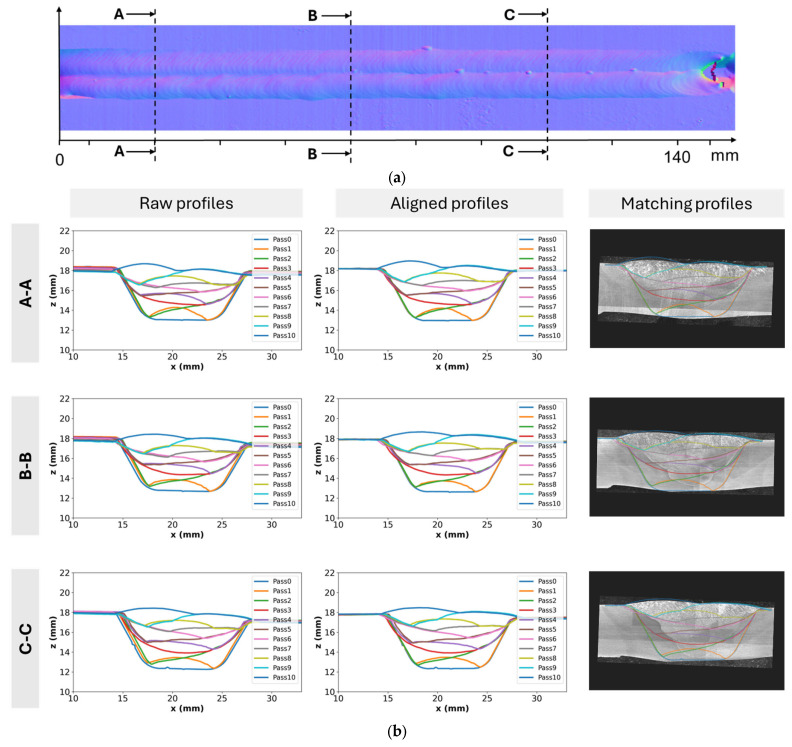
Multi-pass cross-section modelling results: (**a**) normal map of cap pass and three cross-section positions; (**b**) raw profiles, aligned profiles, and matching profiles at three cross-section positions, respectively.

To demonstrate the performance of the welding cross-section model, a welding case with 10 passes along the arc length of 150 mm was conducted. Figure 10a shows a normal map derived from depth data showing the finished bead depth and three cross-section positions in the transverse direction. Each row of Figure 10b is a cross-section of the multi-pass at the A-A, B-B, and C-C positions, respectively. The first column is the raw profiles obtained from the line scanner, the second column is the aligned profiles using our proposed algorithms, and the last column shows the matching between the aligned profiles and real cross-sections. From the raw data, it is clearly shown that the distortion occurred during welding because some profiles did not fit their previous profiles in the groove side, although it was not in the welded positions. The distortion along the *x*-axis ranged from 0.5 to 2 mm in some initial passes. This occurs because the welding process did not heat and cool the material uniformly, causing the welded region to expand and contract much more than the base material. This led to transverse or angular distortion. Moreover, the distortion tended to increase from left (A-A position) to right (C-C position) as it accumulated over time. Especially during the initial root pass, when there was no restraint, significant weld metal shrinkage occurred. While subsequent passes did contribute to some distortion, the overall effect was not cumulative because the earlier passes helped restrain the joint from further movement. In the second column, the proposed algorithm aligned the raw data well while eliminating the deformations. Although the cross-sections were cut and the etching technique was used, it did not clearly show the welding boundaries of each pass. The main reason is that the filler material and the base material were mixed, making it difficult to distinguish the cross-section boundary.

### 4.2. Surface Defect Inspection Model Training Results

To validate the performance of the proposed defect inspection approach, defect data were collected from various experiments. A total of 835 images with defects were divided into two parts, training and validation, with a ratio of 7:3. For a balance between speed and accuracy, two variants of YOLOv10 models were selected for training, including the nano version (YOLOv10-N) and the small version (YOLOv10-S). Instead of training from scratch, transfer learning was employed to customize pre-trained models with the collected data for training. This method can leverage the advantages of trained models as well as massively reduce training costs. Each model was trained for 1000 epochs using the Torch deep learning framework on a single NVIDIA GeForce RTX 4080 GPU, NVIDIA Corporation, Santa Clara, CA, USA, with 16 GB of RAM. For comparison, two types of data with the same labels were used: luminance data converted to grayscale images, and normal maps derived from depth data. Table 4 shows the training results with two metrics, including the mean average precision calculated at the intersection over union (IoU) threshold of 0.5 (mAP50) and the mean average precision calculated over the IoU threshold range of 0.5 to 0.95. Both datasets obtained good results in both models of mAP50 greater than 0.8. Our proposed dataset with a normal map achieved higher accuracy than the luminance dataset in both models at mAP50 values of 0.854 and 0.882, respectively. The YOLOv10-S obtained higher accuracy than the YOLOv10-N model because this variant is more complex. However, it is also a trade-off between the interference speed and accuracy.

**Figure 11:**
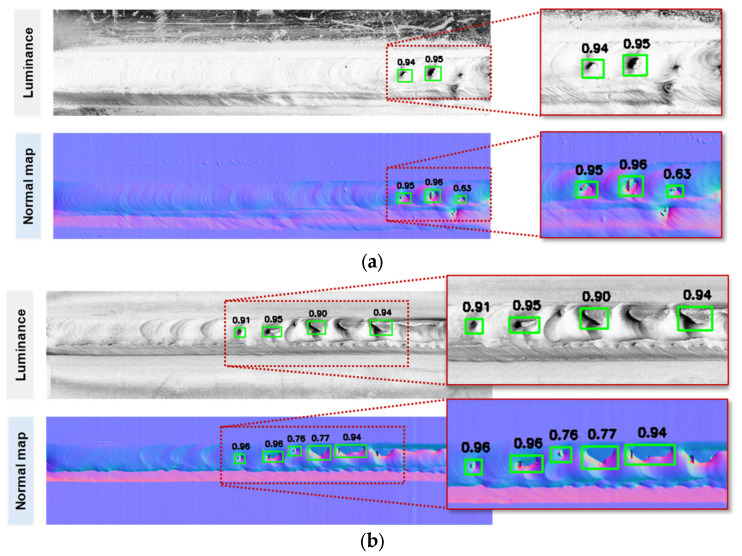
Surface defect inspection results for luminance images and normal map images using YOLOv10-S with different materials: (**a**) SA 106-Gr.B material; (**b**) SUS304 material.

To demonstrate the training results, MLMP welding tests were conducted on two types of materials using models trained with both luminance data and normal maps. The YOLOv10-S models were selected due to their higher training accuracy. Because the input image size was long in the horizontal direction, the sliced technique was used by combining the trained model and the SAHI open-source repository [31]. Figure 11a shows the welding pass of SA 106-Gr.B material with three porosity positions. The score threshold was set at 0.5, where the model trained from normal images can detect three positions, while the model trained from luminance images can only recognize two positions. Similarly, Figure 11b shows the inspection results of the welding pass using SUS304 material, consisting of both porosity and lack of fusion. Most of the defects were detected in the normal image, while the luminance image shows less accuracy. Based on the luminance images, it can be observed that both surface colours are different in the two materials, while the normal images can eliminate the effect of complex surface colour. This point can make the model more effective. In addition, it also needs to increase the amount of data with variety to improve the misrecognition in both images.

### 4.3. Burn-Through Defect Inspection Testing Results

**Figure 12:**
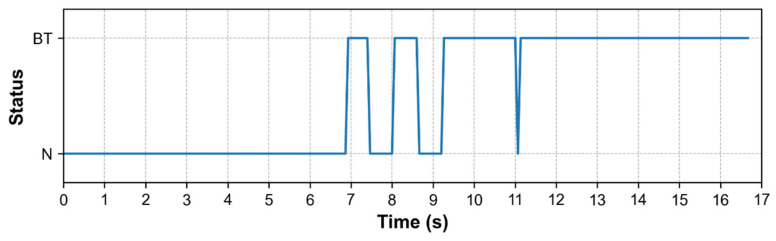
Recognition results of a burn-through test case captured by an IR camera, illustrating the transition from normal status (0–7 s) to burn-through status (7–17 s) over time.

**Figure 13:**
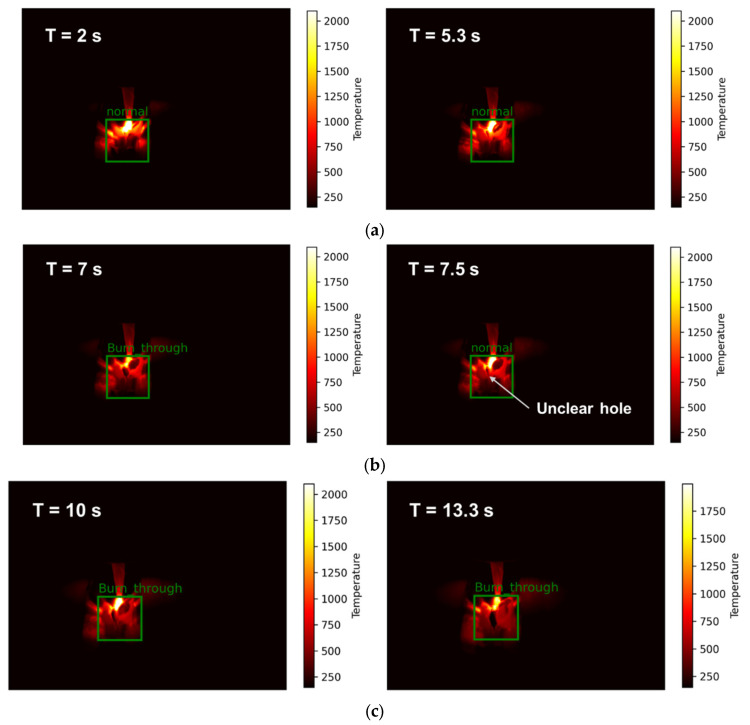
Example results from burn-through defect inspection using an IR camera in each stage: (**a**) normal stage, (**b**) transfer stage normal to burn-through, and (**c**) burn-through stage.

To demonstrate the performance of our proposed algorithm for burn-through defect detection, burn-through defects were intentionally created at a current of 200 A and a voltage of 10.5 V, where the status was controlled to change from normal (N) to burn-through (BT) over time, as shown in Figure 12. Initially, the normal state was almost recognized well before, from the 0th to the 7th second, where an example result is shown in Figure 13a. When the condition was changed to burn-through, the status tended to oscillate for 2 s, as shown from the 7th to the 9th second. This is because when the hole was formed in the early stage, it was not clear and had an influence of arc light, as shown in Figure 13b. After this, the algorithm recognized the burn-through hole well under the electrode tip, as shown in the results in Figure 13c. Based on the timeline, some frames were missing recognition of the burn-through when changing from normal status to burn-through status. However, it did not have much influence on the recognition results because the signal was returned if a high probability of burn-through occurred. Therefore, a counting method was applied to the list of sequence results to determine the status with the highest probability. This technique allowed for reducing the noise during recognition and making the output status more stable.

### 4.4. Integrated System for Weld Bead Monitoring and Surface Defect Detection

**Figure 14:**
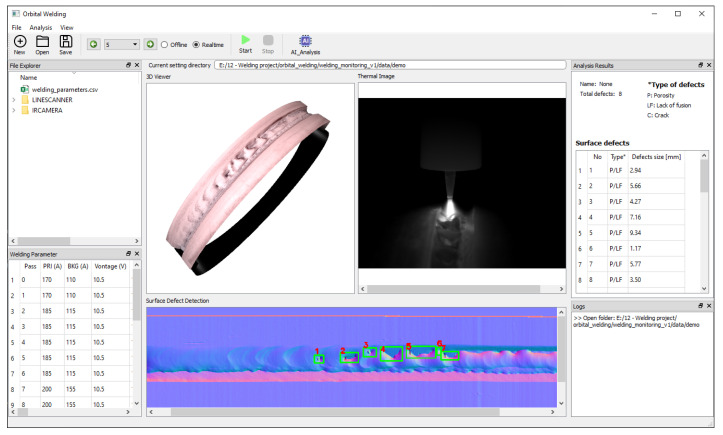
Graphical user interface of the machine vision system for monitoring and surface defect inspection.

For user convenience, a graphical user interface (GUI) was designed to integrate all functions, including welding monitoring and surface evaluation. The interface is shown in Figure 14. There is a recorded data folder, where all welding parameters are shown in “.csv” file format and allowed to be modified by users in the welding parameter dock. In the main window, the 3D viewer and the thermal image widget are used for the visualization of the 3D welded surface and thermal image during welding, respectively. The bottom area shows the surface defect inspection results, and defect sizes are shown on the right-hand side dock. Through this GUI, users can interact with 3D modelling to observe the weld bead or defects in detail. All the data are saved for any further analysis in the future.

## 5. Conclusions

In this paper, a machine system was proposed for monitoring and surface defect inspection of MLMP welding during orbital welding. The system incorporated a groove profile detection algorithm capable of generating accurate cross-sectional representations of multiple weld seams, enabling effective distortion tracking. The novel surface inspection method using normal maps derived from depth data achieved a high mAP50 score of 0.88 for detecting defects such as porosity and lack of fusion, demonstrating robustness against variations in materials. In addition, the proposed burn-through defect detection from the IR image also obtained good accuracy in the testing case, enabling the prompt identification of the critical weld failures. These results confirmed the effectiveness of the proposed system in enhancing weld quality assurance and process control in MLMP welding applications. The following conclusions can be drawn:

‑ By integrating a line scanner and an infrared sensor, this study enabled detailed acquisition and visualization of weld profiles and thermal data during the MLMP welding process.
‑ The robust algorithms were developed for groove profile detection, weld cross-section modelling, and three-dimensional surface reconstruction. These algorithms allowed the precise tracking of welding progress and the quantification of distortion.
‑ The normal maps generated from line scanner depth data were used as input to deep learning models for the evaluation of weld surface quality. This method improved the accuracy of defect detection including porosity and a lack of fusion even in the presence of material surface colour variations.
‑ The image processing algorithm was developed and applied to infrared camera data, enabling the effective real-time detection of burn-through defects during welding. This approach allowed for timely feedback and prompt intervention to prevent critical weld failures.

Limitations and potential work: In this work, the current approach focused on monitoring the welding process and surface defect inspection. This work has the potential to further develop an automated adaptive welding system that can dynamically adjust welding parameters to reduce or eliminate defects. However, while surface defects can be successfully detected, internal defects have not yet been addressed in this work. In addition, it could be further expanded into a comprehensive digital twin model, in which all devices and its motion are digitally represented and synchronized in real time with their physical counterpart. Such a model would enable users to interact with, analyze, and control the system more effectively.

## Figures and Tables

**Table 1:** Specifications of the experimental equipment.

Name	Device	Specification
Welding machine	Welding head: AMI Model M15 Power supply: AMI Model 227 (Arc Machines, Inc., Pacoima, CA, USA)	AVC stroke: 44 mm Wire feed speed: 0–5080 mm/min Travel speed: 2.54–508 mm/min
Line scanner	Keyence LJ-X 8080 (KOREA KEYENCE Co., Ltd., Seoul, Republic of Korea)	Profile data interval: 12.5 μm Profile data count: 3200 points Measure range x: 30–39 mm
IR camera	FLIR A700 (Teledyne FLIR LLC, Seoul, Republic of Korea)	Temperature range: 0–2000 °C Resolution: 640 × 480 pixels

**Table 2:** The tube and groove parameters.

Parameter	Value
Material	SA 106-Gr.B
	SUS 304
Tube diameter	220 mm
Thickness	20 mm
Groove angle	60 degrees

**Table 3:** Hyperparameters adjusted for training.

Argument	Value	Description
epochs	1000	Total number of training epochs
batch	16	Batch size: the number of training examples used in one iteration of model training
imgsz	640	Target image size for training
workers	8	Number of worker threads for data loading
seed	0	Sets the random seed for training, ensuring reproducibility of results across runs with the same configurations
lr0	0.01	Initial learning rate
lrf	0.01	Final learning rate
box	7.5	Weight of the box loss component in the loss function
cls	0.5	Weight of the classification loss in the total loss function

**Table 4:** Model combination experiments on the luminance and normal map dataset.

Model	Dataset	mAP50	mAP50-95
Yolov10-N	Luminance data	0.825	0.445
	Normal map data	**0.854**	**0.491**
Yolov10-S	Luminance data	0.854	0.528
	Normal map data	**0.882**	**0.534**

## Data Availability

The authors declare that the data supporting analyzing and developing the prediction model is not available due to data privacy laws.
